# Psychosocial correlates of adherence self-efficacy and HIV viral suppression among adolescents and young adults in Western Kenya

**DOI:** 10.1371/journal.pone.0341269

**Published:** 2026-02-03

**Authors:** Deepa Oja, Wenwen Jiang, Barbra A. Richardson, Jacinta Badia, James Kibugi, Kristin Beima-Sofie, Sarah Hicks, Jillian Pintye, Molly R. Altman, Kawango Agot, Grace John-Stewart, Pamela Kohler

**Affiliations:** 1 Department of Community Health Systems, School of Nursing, University of California at San Francisco, San Francisco, California, United States of America; 2 Department of Global Health, University of Washington, Seattle, Washington, United States of America; 3 Department of Biostatistics, University of Washington, Seattle, Washington, United States of America; 4 Impact Research and Development Organisation, Kisumu, Kenya; 5 Biobehavioral Nursing and Health Informatics, University of Washington, Seattle, Washington, United States of America; 6 School of Nursing, University of Hawaii, Honolulu, Hawaii, United States of America; 7 Department of Epidemiology, University of Washington, Seattle, Washington, United States of America; 8 Department of Pediatrics, University of Washington, Seattle, Washington, United States of America; 9 Department of Child, Family, and Population Health Nursing, University of Washington, Seattle, Washington, United States of America; South African Medical Research Council, SOUTH AFRICA

## Abstract

**Objective:**

To measure adherence self-efficacy (ASE) to antiretroviral therapies (ART) and evaluate the relationship between ASE, depression, perceived social support, and HIV viral suppression among adolescents and young adults living with HIV (ALHIV) in Kenya.

**Design:**

Cross-sectional analysis of baseline data from a longitudinal cohort of Kenyan ALHIV.

**Methods:**

ALHIV were recruited from nine health facilities in western Kenya. Participants completed behavioral surveys at enrollment, and study team members extracted viral load data from a national database. ASE was assessed using a modified HIV-Adherence Self-efficacy Assessment Survey (HIV-ASES), and depression was assessed as a score of ≥ 10 using the PHQ-9. Linear mixed effects regression modeling and general linear mixed effects regression modeling, clustering by facility, were used to determine associations between ASE scores, viral suppression, and correlates of interest.

**Results:**

Overall, 987 ALHIV age 15 and older were included in this study, 70% were female, 58% ages 15−19, and 57% were attending or had completed at least secondary school. Ninety-six percent had ASE data, 73% (517/703) were virally suppressed, 90% (888/987) scored 9 or below on the PHQ-9, 47% (460/987) reported high perceived social support, and 65% (645/987) were classified as having orphan status. ALHIV who had moderate-to-severe depression had a mean ASE score that was 13.41 points lower (95% CI: −20.12 – −6.52, *p* < 0.001) than those with none-or-mild depression. Female ALHIV had higher odds of viral suppression (adjusted OR: 1.55, 95%CI: 1.07–2.25, *p* = 0.02) as did ALHIV with higher social support (adjusted OR: 1.67, 95%CI: 1.17–2.40, *p* = 0.005). There was no significant association identified between ASE and viral suppression.

**Conclusions:**

Emotional well-being and social support are essential to improve adherence self-efficacy and viral suppression among youth living with HIV.

## Introduction

Adherence to antiretroviral treatment (ART) is critical in maintaining HIV viral suppression and improving health outcomes for adolescents and young adults living with HIV (ALHIV). Low ART adherence increases viral replication and disease progression, reducing quality of life and increasing mortality among individuals living with HIV [1]*.* Multiple studies report that ART adherence among ALHIV is significantly lower than adults [2–4], with barriers to adherence across personal, caregiver and health system-related domains, including stigma, forgetfulness, secrecy, ART side effects, inconvenience, and lack of support [3]. Targeting self-efficacy could be a focal point in addressing some of these ART adherence barriers at the individual level while enabling ALHIV to self-manage their HIV.

Self-efficacy is defined as an individual’s belief in their ability to implement a specific behavior or a set of behaviors [4]. Among adolescents and young adults, self-efficacy is related to positive outcomes such as improved academic performance, emotional health, and quality of life [5]. Self-efficacy score measures have been used to predict outcomes of various chronic conditions such as asthma, type 2 diabetes, hypertension, and other chronic health conditions [6–9].

Within the broader construct of self-efficacy, researchers have examined specific concepts of adherence self-efficacy (ASE) relating to treatment plans. ASE is defined as “confidence in one’s ability to adhere to treatment plans” and has been shown to be an important predictor of medication adherence for HIV [10,11]. Among adults living with HIV, ASE is a strong predictor of initiation and maintenance of ART [12,13]; however, there have been few studies of ASE among adolescents on ART in Kenya [14].

Interventions to target ASE as a mechanism to improve ART adherence may be a potential avenue to increase the self-management of HIV for adolescents and youths, however contributing factors of mental health and social support may attenuate these relationships. Thus, in this paper, we measured ASE to ART and evaluated the relationship between ASE, depression, perceived social support, and HIV viral suppression among ALHIV in Kenya.

## Materials and methods

### Study population and design

This study utilized baseline data from Kenyan ALHIV enrolled in the Data-informed Stepped Care (DiSC) to Improve Adolescent HIV Outcomes cohort study [15]. ALHIV 10–24 years of age were recruited from nine HIV care and treatment facilities located in Kisumu, Homabay, and Migori counties, from April 1, 2019, to February 28, 2020. For this analysis, participants aged 15 years and older were included, consistent with prior use and validation of the ASE scale. Data for this analysis were accessed on October 20, 2022, from the DiSC study data manager at the University of Washington.

### Conceptual framework

**Fig 1:**
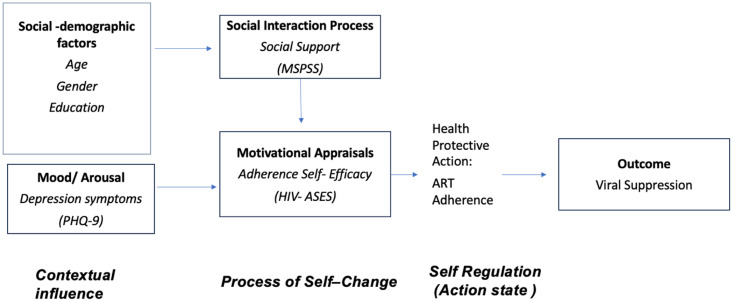
Organization of Study Constructs and Measures Based on the Adaptation of Social Action Theory.

The analysis is guided by CK Ewart’s Social Action Theory (SAT), which emphasizes a model of behavior change and the social context and support that assist in achieving and maintaining that behavior change [16–18]. According to SAT, health behaviors result from the interaction of three domains: *contextual influences*, the *process of self-change,* and *self-regulation* [18]. The *contextual influences* encompass the context in which the health behavior occurs, such as individuals’ background and demographics, life stressors, and mental health state. The *process of self-change* includes self-change processes such as self-efficacy and social support. *Self-regulation* is an action state, which includes actions such as adherence to ART to reduce HIV viral load. Thus, intended health behaviors are shaped by the social-environmental system and interpersonal factors, which can both hinder or facilitate a behavior change [16]. SAT has been utilized in various studies to promote healthy behaviors such as ART adherence [19,20]. Fig 1 represents measures assessed in this study based on SAT.

### Data collection

Behavioral surveys were administered by trained study staff via tablets at enrollment. Viral load (VL) data were obtained from the National AIDS and STI Control Programme database. VL collected within three months before or after completion of the DiSC baseline survey was used for the analysis. If there were two VLs collected within a three-month window, the VL collected closest to the survey date was used.

### Measures

The outcomes in this study were ASE score, measured by the HIV-Adherence Self-efficacy Assessment Survey (HIV-ASES) [21], and VL suppression. HIV-ASES is a psychometric scale that has been previously validated to assess people living with HIV’s confidence to carry out important behaviors around adherence to treatment plans [14,21,22]. In this study of adolescents, we omitted one of the twelve ASES questions, “How confident have you been that you get something positive out of your participation in treatment, even if the medication you are taking does not improve your health?” citing concern that the question would be interpreted as stating ART does not improve health. We categorized participants as having high ASE if the HIV-ASES total score was above 90 [14]. VL suppression was defined as having < 200 copies of HIV RNA/ ml of blood based on Kenya’s HIV Treatment Guideline definition [23].

Predictors analyzed in this study were age groups, sex, education level, orphan status, depression (using the Patient Health Questionnaire-9 (PHQ-9) [24]), and social support (using the Multidimensional Scale of Perceived Social Support (MSPSS) [25]). Orphan status was defined based on the participant’s report of having lost one or both parents. In alignment with clinical guidelines, a PHQ-9 score of 9 or below was considered as having none to mild depressive symptoms, and a score of 10 or higher was considered as moderate to severe depressive symptoms [24]. The MSPSS is a brief questionnaire designed to measure perceptions of social support from three sources: family, friends, and significant others. In this study, high perceived social support was categorized as those with a score above median while low perceived social support were classified as those with a score below median.

### Statistical analysis

Cumulative ASE scores were described in medians with interquartile ranges (IQR) and proportions of viral suppression were described in counts and proportions. Correlates were identified using linear mixed effects regression models and general linear mixed effects regression models, clustered by facility. Variables with *p*-values <0.10 in the bivariate analyses were included in multivariable models for ASE and viral suppression. All analyses were done in R [26].

ASE scores were missing for 4% of participants. We compared participants with and without ASE data across key predictors including age, gender, depression and perceived social support score. Preliminary analyses showed no significant differences in key predictors between participants missing ASE data vs. without, suggesting that the ASE data were likely missing completely at random.

### Ethical approval

We obtained approvals from the University of Washington Institutional Review Board (STUDY00005767) and Maseno University Ethics Review Committee (ERC) (MSU/DRPI/MUERC/00799/19). Caregivers and adolescents provided informed consent and assent until August 2019. On August 22, 2019, we received approval from Maseno ERC to waive parental consent of adolescents ages 15–17 who came to the clinic without a caregiver.

## Results

### Participant characteristics

Survey data from 987 ALHIV ages 15 and older were included in this analysis. The median age of participants was 18 years (IQR:16–21) (Table 1). Most participants (70%) were female. Five hundred and sixty-four participants (57%) currently attended or had completed secondary school. Approximately 90% reported no or mild depressive symptoms over the past two weeks. Most participants (65%) reported that one or both parents were deceased. ASE cumulative scores were relatively high, with a median score of 99 out of 110 (IQR: 90–105). The median MSPSS score was 43 out of 60 (IQR: 38–48).

**Table 1:** Characteristics of ALHIV Participants (n = 987).

Characteristic	Median (IQR)^d^ or N (%)
Age (overall)	18 (16, 21)
15–19 years	576 (58.4)
20–24 years	410 (41.5)
Sex	
Male	296 (30)
Female	690 (70)
Education level	
Primary school	423 (42.9)
Secondary level or higher	564 (57.1)
Cumulative PHQ-9^a^ score	2 (0 - 4)
Depressive symptoms	
None to mild depressive symptoms (PHQ score of ≤9)	888 (90)
Moderate to severe depressive symptoms (Total PHQ score ≥10)	26 (2.6)
HIV adherence self-efficacy scale (ASES)^b^ score	99 (90 - 105)
Multidimensional Scale of Perceived Social Support (MSPSS)^c^ score	43 (38 - 48)
Low perceived social support	470 (46)
High perceived social support	460 (47)
Orphan status	
Yes (one or both parents deceased)	334 (34)
No (both parents are alive)	645 (65)
Viral suppression*	
Suppressed VL^e^ (<200 copies/ml)	517 (73.5)
Non- suppressed VL (≥200 copies/ ml)	186 (26.5)

*among 703 participants with VL data.

Note:

^a^PHQ-9: Personal Health Questionnaire-9.

^b^HIV ASE: HIV HIV-Adherence Self-efficacy Assessment Survey.

^c^MSPSS: Multidimensional Scale of Perceived Social Support.

^d^IQR: Interquartile range.

^e^VL: Viral Load.

### Itemization of the HIV adherence self-efficacy scale

When we evaluated each of the 11 HIV-ASES scale questions in an itemized manner, we found that for all questions, except one, the median score was 10. Question three assessed incorporating treatment in daily routine, including in front of people who don’t know their HIV status, and the median score was 5 (IQR: 0–10) (Table 2).

**Table 2:** Itemized HIV Adherence Self-Efficacy (HIV-ASES) Scale.

HIV-ASES Question	Median score (IQR)
1. Keep to your treatment plan even when side effects begin to interfere with daily activities	10 (8 −10)
2. Include your treatment into your daily routine	10 (9 - 10)
3. Include your treatment into your daily routine even if it means taking medication or doing other things in front of people who don’t know you are HIV-infected	5 (0 - 10)
4. Keep to your treatment schedule even when your daily routine is disrupted	10 (9 - 10)
5. Keep to your treatment schedule when you aren’t feeling well	10 (9 - 10)
6. Keep to your treatment schedule when it means changing your eating habits	10 (9 - 10)
7. Keep with your treatment even if doing so interferes with your daily activities	10 (9 - 10)
8. Keep with the treatment plan your physician prescribed even if your T-cells drop significantly in the next three months	10 (10 − 10)
9. Keep with your treatment even when you are feeling discouraged about your health	10 (9 - 10)
10. Keep with your treatment even when getting to your clinic appointments is a major hassle	10 (9 - 10)
11. Keep with your treatment even when people close to you tell you that they don’t think that it is doing any good	10 (10 − 10)

### Correlates of HIV adherence self–efficacy

We evaluated the association between the cumulative ASE score and risk factors, including age groups, sex, education levels, depressive symptoms, orphan status, and perceived social support (Table 3). In linear mixed effects models clustered by facility, ALHIV who had moderate-to-severe depression had a mean ASE score that was 13.41 points lower (95% CI: – 20.12 – −6.52, *p* < 0.001) than those with none-or-mild depression. There were no statistically significant differences in cumulative ASE scores by age group, sex, education, orphan status, and those with higher than median and lower than median levels of perceived social support in a multivariate analysis.

**Table 3:** Correlates of Adherence Self-Efficacy (n = 1033).

	HIV-ASES score^a^Mean (SD)	Bivariate Analysis^b^ Coefficient estimate (95% CI)	Unadjusted p-value	Multivariate Analysis^c^ Coefficient estimate (95% CI)	Adjusted p-value
**Age group**					
15–19 years	92.2 (18.7)	Ref	0.02	Ref	
20–24 years	95.6 (16.9)	2.91(0.58–5.30)		2.06 (−0.26–4.44)	0.08
**Sex**					
Male	91.1 (20.1)	Ref		Ref	
Female	94.7 (17.0)	3.27 (0.76 - 5.83)	0.01	1.78 (−0.75–4.32)	0.17
**Education level**					
Primary school	94.3 (18.3)	Ref			
Secondary school	93.2 (17.9)	−1.05 (−3.41 - 1.30)	0.38		
**Perceived social support** ^**d**^					
Low perceived social support	94.7 (16.6)	Ref			
High perceived social support	93.3 (18.4)	−1.67 (−3.97–0.67)	0.15		
**Depression symptoms** ^**e**^					
None-mild depression	94.8 (16.3)	Ref		Ref	< 0.001
Moderate-severe depression	82.4 (30.4)	−13.56 (−20.30 – −6.67)	<0.001	−13.41 (−20.12 - −6.52)	
**Orphan Status**					
No (both parents alive)	94.1 (16.8)	Ref			
Yes (one or both parents deceased)	93.4 (18.7)	−0.20 (−2.66–2.20)	0.87		

^a^Total mean HIV adherence self-efficacy scale (HIV-ASES) score. Total cumulative score possible was 110. Mean score among all participants was 93.6 and median was 99.

^b^Bivariate linear mixed effects model clustered by facility to account for random effect.

^c^Multivariable linear mixed effects model clustered by facility to account for random effect. Only variables with p ≤ 0.10 in bivariate analysis were used in the multivariate model.

^d^Multidimensional Scale of Perceived Social Support (MSPSS) measured perceived social support. High social support was defined as score greater than median MSPSS score. Median MSPSS score was 43. Total cumulative score possible was 60.

^e^Patient Health Questionnaire-9 (PHQ-9) assessed depression symptoms. PHQ-9 score of ≤9 or below was considered as having none to mild depressive symptoms and a score of ≥10 was considered as moderate to severe depressive symptoms.

### Correlates of viral suppression

A total of 703 (71%) ALHIV had viral load data within 3 months of baseline surveys, and 74% (*n* = 517) were virally suppressed. ALHIV with higher than median social support score had higher odds of viral suppression compared to those with lower than median social support score (78% vs. 68% respectively, adjusted OR [aOR]: 1.67, 95%CI:1.17–2.49, *p* = 0.009) (Table 4). Additionally, female ALHIV had higher odds of viral suppression (76%) than male ALHIV (68%) (aOR:1.55, 95%CI: 1.07–2.25, *p* = 0.02). Age groups, education levels, severity of depression, and ASE scores were not associated with viral suppression in univariate regression models.

**Table 4:** Correlates of HIV Viral Suppression (VL < 200).

	Viral suppression^a^ (N, %)	Bivariate Analysis^b^ Odds Ratio (95% CI)	Unadjusted p-Value	Multivariate Analysis^c^ Odds Ratio (95% CI)	Adjusted p-value
**Age groups**					
15–19 years (n = 437)	315 (72)	Ref			
20–24 years (n = 266)	202 (76)	1.12 (0.77–1.61)	0.55		
**Sex**					
Male (n = 232)	157 (68)	Ref		Ref	
Female (n = 471)	360 (76)	1.5(1.05–2.15)	0.02	1.55 (1.07–2.25)	0.02
**Education level**					
Primary school (n = 302)	220 (73)				
Secondary school (n = 401)	297 (74)	1.0(0.92–1.1)	0.93		
**Perceived social support** ^**d**^					
Low (n = 334)	226 (68)	Ref		Ref	
High (n = 327)	255 (78)	1.67 (0.17–2.4)	0.005	1.67 (1.17–2.4)	0.005
**Depression symptoms** ^**e**^					
None-mild depression (n = 628)	457 (73)	Ref			
Moderate- severe depression (n = 18)	16 (88)	3.13 (0.7–14.02)	0.14		
**Adherence self-efficacy** ^**f**^					
Low (n = 351)	254 (72)	Ref			
High (n = 323)	238 (74)	0.99 (0.7–1.4)	0.94		

^a^Viral suppression classification is based on Kenya’s HIV Treatment Guideline definition and cut-off values for suppressed status as < 200 copies of HIV RNA/ ml of blood

^b^Bivariate generalized linear mixed effects regression model accounting facility cluster as a random effect.

^c^Multivariate generalized linear mixed effects regression models accounting facility cluster as a random effect. Only variables with p ≤ 0.10 in bivariate analysis were used in the multivariate model.

^d^Multidimensional Scale of Perceived Social Support (MSPSS) measured perceived social support. High social support was defined score greater than median MSPSS score. Median MSPSS score was 42 among 703 ALHIV with matching VL. Total cumulative score possible was 60.

^e^Patient Health Questionnaire-9 (PHQ-9) assessed depression symptoms. PHQ-9 score of ≤9 or below was considered as having none to mild depressive symptoms and a score of ≥10 was considered as moderate to severe depressive symptoms.

^f^HIV adherence self-efficacy scale (ASE) measured adherence self-efficacy. High adherence self -efficacy was defined as score above the median ASE score. Median ASE score was 99 among 703 ALHIV with matching VL. Total cumulative score possible was 110.

## Discussion

This cross-sectional analysis of a large cohort of ALHIV found a positive correlation between adherence self-efficacy and mental health and an association between social support and viral suppression. Our findings indicate relatively high levels of adherence self-efficacy to ART among ALHIV, with a single item driving differences in ASE scores: incorporating treatment into a daily routine even if it meant taking medication in front of others who were unaware of their HIV status. Similar findings of difficulty taking medication in public spaces, using the HIV-ASES psychometric tool, have been reported among ALHIV in Nairobi [27,28]. Perceived stigma and fear of unintended disclosure associated with taking ART in public have been noted among ALHIV globally as contributors to ART adherence [29–31]. Well-designed interventions that target internal stigma reduction and enable adolescents to take their ART in whatever spaces they choose are imperative to improve HIV outcomes among ALHIV.

Our findings indicate that depression is associated with reduced ASE scores. Consistent with our findings, other studies have also observed that depression is linked to lower levels of ART adherence among individuals living with HIV [32–34], and further, self-efficacy has been found to mediate the association between depression and treatment adherence in chronic illnesses such as heart failure and hypertension [11,35]. Identifying and treating mental health conditions such as depression potentially impacts self-efficacy skills, which leads to increased medication adherence and, ultimately, improved HIV outcomes among ALHIV. Thus, at the health systems level, structural interventions, such as prioritization of treatment for mental health conditions, which target emotional and physiological states, could be utilized to enhance self-efficacy for HIV management.

Social support has similarly been noted to have a robust effect on health, with its role as a protective factor against morbidity and all-cause mortality [36,37]. SAT and Social Cognitive Theory both indicate that social support likely influences self-efficacy beliefs [18,38,39]. However, we did not find that the perceived social support score was associated with the adherence self-efficacy score, though other studies have found that social support had a positive relationship with treatment self-efficacy [39,40]. Similar to other studies, we found that ALHIV with high perceived social support had higher odds of suppressed viral loads [41,42]. This suggests that social support plays a critical role in health outcomes, potentially through increased motivation, emotional well-being, or practical support that facilitates treatment adherence. Thus, these findings underscore the importance of a strong support network for ALHIV to achieve viral suppression and improve health outcomes.

Our study had generally high self-efficacy scores and viral suppression and may not have had sufficient variability or statistical power to detect the impact of self-efficacy on viral suppression. Other studies have shown that ART adherence self-efficacy has been associated with increased self-reported adherence and VL suppression among ALHIV in Kenya and among adults in the United States [14,43]. Studies of adolescents and youth in Thailand report that adherence self-efficacy is associated with increases in overall quality of life and physical health [28]. Given self-efficacy’s mediating role on SAT and based on literature findings as discussed above, it is a potential target area to increase self-management of HIV for adolescents and youth.

This study has strengths and limitations. This analysis was a cross-sectional study thus, temporal changes in variables were not assessed. Participants of our study were adolescents and youth in western Kenya and may not be generalizable to the broader population. Further, participants filled out a lengthy survey that consisted of multiple measures and could have been impacted by participant fatigue. ALHIV were also not required to complete all the survey questions; thus, those with missing data may have different experiences or opinions.

## Conclusion

Emotional well-being and social support are essential to improve adherence, self-efficacy, and viral suppression among ALHIV. To meet viral suppression goals in ALHIV, interventions targeting perceived stigma reduction and increasing self-efficacy may empower and enable adolescents and youths to take their HIV medication in any space they desire.
